# Circadian rhythm of autonomic activity in non diabetic offsprings of type 2 diabetic patients

**DOI:** 10.1186/1475-2840-4-15

**Published:** 2005-10-01

**Authors:** A Fiorentini, A Perciaccante, A Paris, P Serra, L Tubani

**Affiliations:** 1 III Clinica Medica, Department of Clinical Medicine, University "La Sapienza", Rome, Italy; 2 Medicina Interna E, Department of Clinical Medicine, University "La Sapienza", Rome, Italy

**Keywords:** heart rate variability, circadian rhythm, insulin resistance

## Abstract

The aim of the present study was to evaluate, by heart rate variability (HRV) with 24-hours ECG Holter (HRV), the circadian autonomic activity in offspring of type 2 diabetic subjects and the relation with insulin-resistance. METHODS: 50 Caucasian offsprings of type 2 diabetic subjects were divided in two groups: insulin-resistant offsprings (IR) and non insulin-resistant offsprings (NIR). Autonomic nervous activity was studied by HRV. Time domain and spectral analysis (low frequency, LF, and high frequency, HF, provide markers of sympathetic and parasympathetic modulation when assessed in normalized units) were evaluated. RESULTS. Time domain showed a reduction of total SDNN in IR (p < 0.001) and NIR (p 0.047) versus controls. Spectral analysis showed a total and night LF higher in IR and NIR than in control group (all p < 0.001). CONCLUSION. In frequency domain, the analysis of sympathetic (LF) and parasympathetic (HF) component evidenced an association between the offspring of type 2 diabetic subjects and a sympathetic overactivity. A global reduction and alteration of circadian rhythm of autonomic activity are present in offspring of type 2 diabetic patients with and without insulin resistance. The data of our study suggested that an autonomic impairment is associated with the familiarity for type 2 diabetes independently to insulin resistance and that an impairment of autonomic system activity could precede the insulin resistance.

## Background

Sympathetic and parasympathetic components of neurovegetative system regulate cardiac activity. Spectral analysis of heart rate variability (HRV) is a non invasive metodic used to assess cardiac autonomic activity. The autonomic activities can be assessed in HRV by the relative distribution (evaluated in normalized units) of low frequency (LF), as an index of sympathetic modulation and of high frequency (HF), as an index of parasympathetic modulation. The analysis of HRV has been used for evaluate the autonomic cardiac activity in numerous pathophisyologic conditions [1-9]: an impaired heart rate variation is a marker of autonomic neuropathy [10,11] as in diabetic subjects [12-16].

Several studies have showed a sympathetic overactivity also in non diabetic insulin resistant group [17-19]. However, little is known about the association between the familiarity for type 2 diabetes mellitus and the autonomic activity. An increased LF/ HF ratio (low frequency/high frequency ratio) has identified in only insulin resistant offsprings of type 2 diabetic subjects [20]. De Angelis et al [21] has showed this condition at rest but not under stimulated conditions. Laitinen et al [22] has identified a sympathetic overactivity during acute hyperinsulinemia both in insulin resistant and non insulin resistant offsprings of type 2 diabetic subjects.

The aim of the present study was to evaluate, by heart rate variability with 24-hours ECG Holter registration, the autonomic activity and circadian autonomic rhythm in offspring of type 2 diabetic subjects and to evaluate the possible impact of sympathetic activity on insulin resistance. We tested the hypothesis that in non insulin resistant offsprings of type 2 diabetic subjects an alteration of circadian rhythm of autonomic activity are present and that the impairment of autonomic system activity could precede the insulin resistance.

## Subjects and methods

70 consecutive caucasian offsprings of type 2 diabetic subjects, admitted to our department, were screened. In all subjects, after an overnight fast, oral glucose tolerance tests (OGTTs) was performed: samples blood for glucose and plasma insulin were collected before and 2 h after a glucose load consisting of 75 g glucose anhydrate in 300 ml of water ingested over the course of 5 min. Also, fasting plasma insulin was measured to evaluate the insulin-resistance by the homeostasis model assessment-index (HOMA-I). Among them, 50 caucasian subjects (age: 47.71 ± 9.96 years, 32 men and 18 women), with normal OGTTs, were admitted in this study and were divided in two groups: offsprings with insulin resistance and offsprings without insulin resistance:

Subjects with hypertension [23], diabetes mellitus, impaired fasting glycemia, impaired glucose tolerance [24], obesity, dyslipidemia, cardiac arrhythmias, microalbuminuria and with drug treatment or diseases that could potentially disturbs carbohydrate metabolism (glucocorticoids, furosemide, beta-blockers, etc.) and cardiac autonomic activity (beta-blockers, anti-arrhythmics, ACE-inhibitors) were excluded.

The control group consisted of 25 sex and age matched healthy non insulin resistant subjects with normal OGTTs and without familiarity for type 2 diabetes mellitus.

Height, weight and body circumferences were measured on all subjects. Body mass index (BMI, kg/m^2^) was calculated as weight divided by height squared. Waist-to-hip ratio (WHR) was defined as waist circumference divided by hip circumference.

Informed consent was obtained from all participants; all the investigations were performed in accordance with the participants of the Declaration of Helsinki.

### Insulin resistance

The insulin-resistance was evaluated by the homeostasis model assessment index (HOMA-I) [25-28]. The HOMA-I was calculated by the formula: fasting plasma glucose (mmol/L) x fasting plasma insulin (μU/ml)/ 22,5, as described by Matthews and coworkers [29]. Insulin-resistance was defined as the third and fourth quartiles of HOMA-I.

The index subject were subdivide into two groups based on HOMA-I: 1) group of insulin-resistant offsprings (IR); 2) group of non insulin-resistant offsprings (NIR).

### HRV assessment

Autonomic nervous activity was evaluated by heart rate variability (HRV) analysis during 24-hour ECG recording. All Holter recordings were performed using a three-channel recorder. Autonomic nervous activity was analysed following the recommendations of the Task Force of the European Society of Cardiology and the North American Society of Pacing and Electrophysiology [30]. Spectral estimates of R-R interval were obtained from stationary regions free of ectopic beats and technical artefacts. The standard deviation of normal-to-normal RR intervals (SDNN) (ms) and the square root of the mean of the sum of the squares of differences between adjacent NN intervals (RMS-SD), correlated with parasympathetic system, were calculated and were divided in two periods: night (0 am – 6 am) and day (7 am – 9 pm). Fast Fourier Transform was used to obtain power spectral estimates of HRV. Total power in the frequency range (0 – 0.40 Hz) was divided into low frequency (LF: 0.04 – 0.15 Hz, modulated by sympathetic system) and high frequency (HF: 0.15 – 0.40 Hz, modulated by parasympathetic system). The power of LF and HF components was considered in normalized units (n.u.). Subjects were analysed for 24 hours, at 10 minutes interval. Artificial data and arrhythmic were excluded. The day was divided in four periods: night (0 am – 6 am), morning (7 am – 12 am), afternoon (1 pm – 6 pm), evening (7 pm – 11 pm). Data analyses were performed with software Del Mar Avionics Accuplus 363, Irvine California, USA.

### Statistical analysis

All analysis were done with SPSS 12.0 (SPSS Inc., Chicago, IL, USA) for Windows XP. Data are presented as means + SD. For data with multiple time points, variables were analysed by the general linear model ANOVA and simple regression analyses were carried out by standard techniques 95% confidence intervals (CT) were calculated for regression coefficient. Means values were considered significant at p < 0.05.

## Results

### Clinical characteristics

The groups had not statistically significant difference in age, sex and anthropometrics parameters (i.e. BMI, waist and hip circumference, waist to hip ratio, table 1).

**Table 1:** Clinical characteristics for each study-group of offsprings and controls.

	IR	**NIR**	**Controls**	**p Value**
**Sex (M/F)**	16/9	18/7	16/9	
**Age (years)**	49,12 ± 6,68	49,80 ± 6,53	53,69 ± 3,72	0,190
**BMI (kg/m2)**	25.41 ± 4.67	25.50 ± 3.69	25.09 ± 2.23	0,120
**Glycemia '0 (mg/dl)**	93,375 ± 11,06	99,66 ± 1,30	94,00 ± 6,15	0,095
**Glycemia '120 (mg/dl)**	97,62 ± 17,45	90,00 ± 26,60	92,66 ± 18,22	0,095
**Fasting plasma insulin (uU/ml)**	17,95 ± 6,35	7,66 ± 0,50	7,16 ± 0,21	0,000
**HOMA index**	4,41 ± 0,80	1.83 ± 0,12	1,45 ± 0,25	
**SP (mmHg)**	123,20 ± 3,70	122,60± 6,35	120.20 ± 7.09	0,013
**DP(mmHg)**	80,00 ± 3,16	79,80± 3,56	78.80 ± 4.90	0,014
**HR (bpm)**	74.75 ± 4.57	70.25 ± 4.11	68.50 ± 5.92	0,011

SP: systolic pressure, DP: diastolic pressure, HR: heart rate, BMI: body mass index, HOMA-I: the homeostasis model assessment-index, IR: insulin resistant offsprings of type 2 diabetic patients, NIR: non insulin resistant offsprings of type 2 diabetic patients.

### Heart rate variability

Tables 2 and 3 show the means of autonomic function measures for each group.

**Table 2:** Means (95%CI) RR intervals in the different time periods for each offspring group and controls.

R-R (msec)	IR	NIR	Controls
Night (0–6 am)	895,39 (821,61–969,17)	873,36 (781,09–965,64)	1126,75 (1060,44–1193,05)
morning (7–12 am)	792,44 (741,58–843,29)	752,26 (719,55–784,98)	870,29 (805,43–935,15)
afternoon (1–6 pm)	690,28 (658,75–721,82)	689,16 (638,25–740,07)	749,54 (615,63–883,46)
evening (7–11 pm)	734,37705,02–763,72)	596,87 (360,36–833,39)	910,82 (808,78–1012,87)

Value are means (95% CI), IR: insulin resistant offsprings of type 2 diabetic patients, NIR: non insulin resistant offsprings of type 2 diabetic patients, RR: normal-to-normal R-R interval (ms)

**Table 3:** Autonomic function measures for each offspring group and controls.

	**IR**	**NIR**	**Controls**	**P value**
**SDNN (msec)**	86.10 ± 35.96	119.70 ± 28.22	131.90 ± 29.52	0,000
**SDNN day (msec)**	105.21 ± 28.37	128.77 ± 30.60	120.29 ± 26.97	0,062
**SDNN night (msec)**	118.99 ± 46.16	112.40 ± 7.54	119.57 ± 38.25	0,822
**RMS-SD (msec)**	43.15 ± 20.60	42.63 ± 9.18	36.75 ± 10.59	0,331
**RMS-SD day (msec)**	41.58 ± 18.64	34.77 ± 5.75	31.37 ± 9.07	0,050
**RMS-SD night (msec)**	47.47 ± 28.80	55.70 ± 18.46	47.47 ± 10.60	0,485
**LF u.n.**	70.88 ± 6.20	67.59 ± 4.46	55.46 ± 7.49	0,000
**HF u.n.**	23.63 ± 6.67	27.09 ± 3.74	37.65 ± 7.53	0,000
**LF/HF**	3.34 ± 1.37	3.72 ± 0.72	2.44 ± 1.09	0,004
**LF night u.n.**	3.34 ± 1.37	3.72 ± 0.72	2.44 ± 1.09	0,000
**HF night u.n.**	25.72 ± 8.19	36.53 ± 3.17	58.62 ± 6.59	0,000
**LF/HF night**	3.06 ± 1.49	2.21 ± 0.54	0.79 ± 0.48	0,000
**LF u.n. morning**	73.45 ± 9.61	69.04 ± 7.41	69.54 ± 8.36	0,298
**HF u.n. morning**	22.12 ± 7.17	24.30 ± 8.52	23.05 ± 7.13	0,765
**LF/HF morning**	4.15 ± 2.80	5.69 ± 3.11	3.70 ± 1.72	0,122
**LF u.n. afternoon**	70.59 ± 10.62	70.06 ± 4.26	70.09 ± 6.62	0,979
**HF u.n. afternoon**	23.21 ± 10.25	21.42 ± 3.79	22.30 ± 5.09	0,794
**LF/HF afternoon**	4.65 ± 1.71	3.74 ± 0.46	3.67 ± 1.30	0,647
**LF u.n. evening**	71.90 ± 10.70	71.90 ± 10.70	56.75 ± 7.75	0,000
**HF u.n. evening**	23.52 ± 6.28	24.28 ± 9.08	35.75 ± 9.14	0,001
**LF/HF evening**	3.30 ± 1.00	4.12 ± 2.21	2.00 ± 0.88	0,009

Value are means ± SD, IR: insulin resistant offsprings of type 2 diabetic patients, NIR: non insulin resistant offsprings of type 2 diabetic patients, SDNN: standard deviation of all sinus rhytm RR intervals, RMS SD: the square root of the mean of the sum of the squares of differencies between adjacent NN intervals, LF: low frequence, HF: high frequence, hours of Holter registration: night time = 0–6 am, morning = 7–12 am, afternoon = 1–6 pm, evening = 7–11 pm. P Value is between groups.

### Time domain

**Figure 1:**
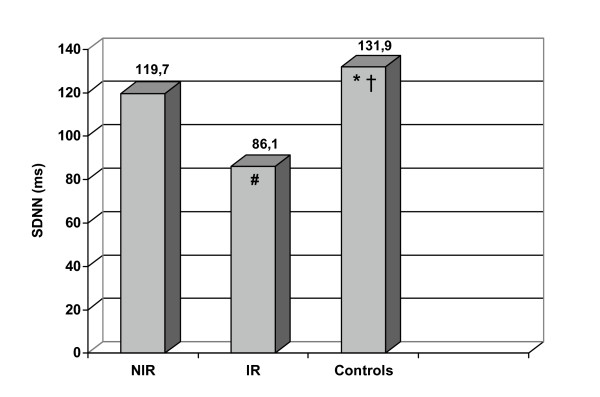
**SDNN value in offsprings of type 2 diabetic patients and controls. **SDNN: standard deviation of all sinus rhythm RR intervals, IR: insulin resistant offsprings of type 2 diabetic patients, NIR: non insulin resistant offsprings of type 2 diabetic patients. #p:0.003IR vs NIR; *p:0.047 controls vs NIR and † p:0.001 controls vs IR

Time domain analysis of HRV showed a reduction statistically significant of total SDNN in IR (p < 0.001) and NIR (p 0.047) groups (IR: 86.10 ± 35.96 ms, NIR: 119.70 ± 28.22 ms) versus control group (131.90 ± 29.52 ms) (fig. 1). Total SDNN were reduced in IR group when compared with (NIR) NIR group (p 0.003). These results showed a total activity reduction of autonomic system in insulin resistant and non insulin resistant offsprings of type 2 diabetic patients. The autonomic activity reduction is major in IR group than NIR group.

RMS-SD in night time not increased in IR group. Therefore insulin-resistance was associated with alteration of circadian rhythm of parasympathetic component (at the night).

### Frequency domain

Frequency domain analysis of HRV showed a total LF higher (all p < 0.001) in IR (70.88 ± 6.2 n.u.) and NIR (67.59 ± 4.46 n.u.) groups than in control group (55.46 ± 7.49 n.u.). No difference was noticed between IR and NIR groups (p: 0.157). HF were lower in IR (23.63 ± 6.67 n.u) and NIR (27.09 ± 3.74 n.u.) than in control group (37.65 ± 7.53) (all p < 0.001), while not statistically significant difference were between the groups of offsprings type 2 diabetic patients, p: 0.127. LF/HF total is higher in IR (3.34 ± 1.37) and NIR (3.72 ± 0.72) groups versus control group (2.44 ± 1.09), respectively p 0.016 and p 0.002.

#### Night (0 am – 6 am)

LF value are higher in IR (67.80 ± 9.70 n.u.) and NIR (59.60 ± 1.75 n.u.) groups than in control groups (35.21 ± 5.97) (all p < 0.001) and are higher in IR group when compared with NIR group, p 0.001). HF are lower in IR (25.72 ± 8.19 n.u.) and NIR group (36.53 ± 3.17) than in control groups (58.62 ± 6.59) (all p < 0.001 vs controls).

#### Morning (7 am – 12 am)

LF and HF had not statistically difference between IR (LF: 73.45 ± 9.61 n.u.; HF n.u.: 22.12 ± 7.17 n.u.), NIR (LF: 69.04 ± 7.41 n.u.; HF: 24.30 ± 8.52 n.u) and control groups (LF: 69.54 ± 8.36 n.u.; HF: 23.05 ± 7.13 n.u.).

#### Afternoon (1 pm – 6 pm)

LF, HF and LF/HF had not statistically difference between IR (LF: 70.59 ± 10.62 n.u.; HF: 23.21 ± 10.25 n.u.; LF/HF: 4.65 ± 1.71), NIR (LF: 70.06 ± 4.26 n.u.; HF: 21.42 ± 3.79 n.u.; LF/HF: 3.74 ± 0.46) and control groups (LF: 70.09 ± 6.62 n.u.; HF: 22.30 ± 5.09 n.u.; LF/HF: 3.67 ± 1.30).

#### Evening (7 pm – 11 pm)

LF were higher in IR (71.90 ± 10.70 n.u.) and NIR (71.90 ± 10.70 n.u.) groups than control group (56.75 ± 7.75 n.u.) (all p < 0.001). HF were lower in IR (23.52 ± 6.28 n.u.) and NIR (24.28 ± 9.08 n.u.) groups than control group (35.75 ± 9.14 n.u.), respectively p: 0.001 and p: 0.002. LF/HF is higher in IR (3.30 ± 1.00) and NIR (4.12 ± 2.21) groups than control group (2.00 ± 0.88) (respectively p: 0.04 and p: 0.002).

## Discussion

**Figure 2:**
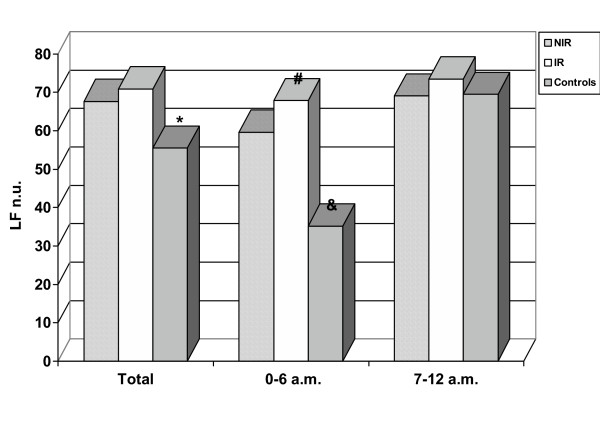
**LF value in offsprings of type 2 diabetic patients and control. **IR: insulin resistant offsprings of type 2 diabetic patients, NIR: non insulin resistant offsprings of type 2 diabetic patients, LF: low frequency, hours of Holter registration: 0–6 am= night time, 7–12 am = morning. * p < 0.001 NIR and IR vs controls, #: p:0.001 IR vs NIR; & p < 0.001 controlsvs NIR and IR.

**Figure 3:**
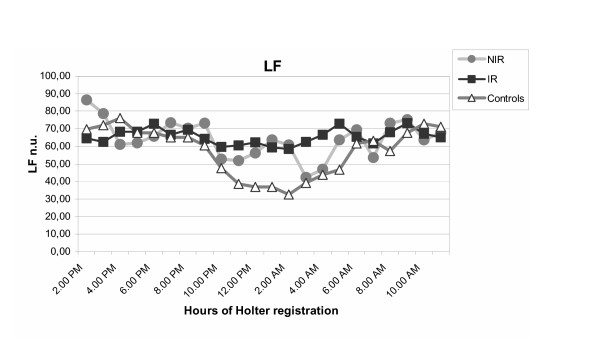
**Circadian variation of LF in IR, NIR and in control group. **Gray circles: group of non insulin resistant offsprings of type 2 diabetic patients; black squares: group of insulin resistant offsprings of type 2 diabetic patients; white triangles: control group; IR: insulin resistant offsprings of type 2 diabetic patients, NIR: non insulin resistant offsprings of type 2 diabetic patients, LF: low frequency.

**Figure 4:**
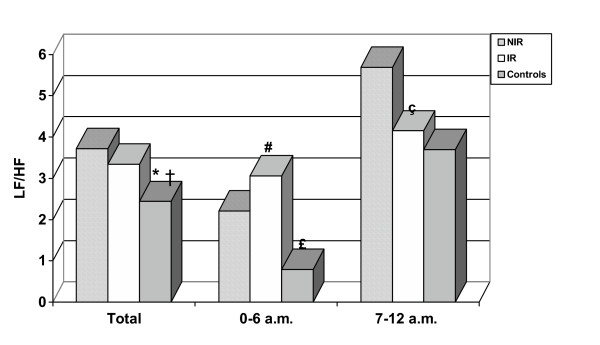
**LF/HF value in offsprings of type 2 diabetic patients and control. **IR: insulin resistant offsprings of type 2 diabetic patients, NIR: non insulin resistant offsprings of type 2 diabetic patients, LF/HF: low frequency/high frequency, hours of Holter registration: 0–6 am= night time, 7–12 am = morning. *p.0.016 controls vs IR; †p:0.002 control vs NIR; # p:0.015 IR vs NIR; £ p:0.0001 controls vs NIR and IR; ç p:0.043 controls vs NIR.

The data of our study suggested that an autonomic impairment is associated with the familiarity for type 2 diabetes independently of insulin resistance. In frequency domain, the analysis of sympathetic (LF) and parasympathetic (HF) component and the symphatovagal balance (LF/HF) evidenced an association between the familiarity and a sympathetic overactivity, especially in nocturnal period, demonstrated by increase of LF (figure 2 and figure 3) and LF/HF ratio (figure 4). This autonomic impairment is major in insulin resistant offsprings than non insulin resistant offsprings of type 2 diabetic patients. Moreover, our study had demonstrated, in time domain analysis of HRV, a significant reduction of the total autonomic system activity in both groups, expressed by progressive decrease of SDNN value from NIR to IR groups. These results indicated that the familiarity of type 2 diabetes mellitus is related to a global reduction of autonomic nervous system and that the dysautonomia increases if offsprings are insulin resistant.

In summary, a global reduction and alteration of circadian rhythm of autonomic activity are present in offspring of type 2 diabetic patients, without and with insulin resistance (figure 2, 3).

A long term observation can answer the question whether autonomic abnormality precede the occurence of insuline resistence and play a role in the complex pathogenesis of the insulin resistance and type 2 diabetes mellitus. Others studies occurs to explain a possible pathogenetic role of autonomic dysfunction in the development of insulin resistance and type 2 diabetes mellitus.

## Limitations

A limitation in this study is the use of the HOMA index as a conventional indicator of insulin resistance. The best method for assessment of insulin resistance is the glucose clamp technique, however, the HOMA model has proved be a robust clinical and epidemiological tool in descriptions of the pathophysiology of diabetes, already quoted in > 500 publications, it has become one of standard tools in the armamentarium of the clinical physiologist [23].

Other study are necessary to determine the mechanism whereby insulin-resistance may be related to autonomic dysfunction.

## List of abbreviations

**BMI**: body mass index; **DM**: type 2 diabetes mellitus; **HF**: high frequency; **HOMA-I**: the homeostasis model assessment-index; **HRV**: heart rate variability; **LF**: low frequency; **OGTTs**: oral glucose tolerance tests; **RMS-SD**: the square root of the mean of the sum of the squares of differences between adjacent NN intervals; **SDNN**: The standard deviation of normal-to-normal RR intervals; **WHR**: waist-to-hip ratio.

## Competing interests

The author(s) declare that they have no competing interests.
